# The Effect of Low Carbohydrate Diet on Polycystic Ovary Syndrome: A Meta-Analysis of Randomized Controlled Trials

**DOI:** 10.1155/2019/4386401

**Published:** 2019-11-26

**Authors:** Xiaoshuai Zhang, Yang Zheng, Yanan Guo, Zhiwen Lai

**Affiliations:** ^1^Chongqing Medical University, No. 1, Medical College Road, Yuzhong District, Chongqing 400010, China; ^2^Department of Gynecology, Chengdu Xinan Gynecological Hospital, Chengdu, China

## Abstract

**Objective:**

To assess the effect of a low carbohydrate diet (LCD) on women with polycystic ovary syndrome (PCOS).

**Methods:**

Data from randomized controlled trials (RCTs) were obtained to perform a meta-analysis of the effects of LCD in PCOS patients. The primary outcomes included the changes in BMI, homeostatic model assessment for insulin resistance (HOMA-IR), and blood lipids, including total cholesterol (TC), low-density lipoprotein cholesterol (LDL-C), and high-density lipoprotein cholesterol (HDL-C), follicle-stimulating hormone (FSH), luteotropic hormone (LH), total testosterone (T), and sex hormone-binding globulin (SHBG).

**Results:**

Eight RCTs involving 327 patients were included. In comparison with the control group, the LCD decreased BMI (SMD = −1.04, 95% CI (−1.38, −0.70), *P* < 0.00001), HOMA-IR (SMD = −0.66, 95% CI (−1.01, −0.30), *P* < 0.05), TC (SMD = −0.68, 95% CI (−1.35, −0.02), *P* < 0.05), and LDL-C (SMD = −0.66, 95% CI (−1.30, −0.02), *P* < 0.05). Stratified analyses indicated that LCD lasting longer than 4 weeks had a stronger effect on increasing FSH levels (MD = 0.39, 95% CI (0.08, 0.71), *P* < 0.05), increasing SHBG levels (MD = 5.98, 95% CI (3.51, 8.46), *P* < 0.05), and decreasing *T* levels (SMD = −1.79, 95% CI (−3.22, −0.36), *P* < 0.05), and the low-fat and low-CHO LCD (fat <35% and CHO <45%) had a more significant effect on the levels of FSH (MD = 0.40, 95% CI (0.09, 0.71), *P* < 0.05) and SHBG (MD = 6.20, 95% CI (3.68, 8.72), *P* < 0.05) than the high-fat and low-CHO LCD (fat >35% and CHO <45%).

**Conclusion:**

Based on the current evidence, LCD, particularly long-term LCD and low-fat/low-CHO LCD, may be recommended for the reduction of BMI, treatment of PCOS with insulin resistance, prevention of high LDL-C, increasing the levels of FSH and SHBG, and decreasing the level of *T* level. Together, the analyzed data indicate that proper control of carbohydrate intake provides beneficial effects on some aspects of PCOS and may represent one of the important interventions improving the clinical symptoms of affected patients.

## 1. Introduction

Polycystic ovary syndrome (PCOS) involves reproductive, metabolic, and hormonal disorders and accounts for 50–70% cases of anovulatory infertility in women of childbearing age [1]. The worldwide prevalence of PCOS is 6–10% and tends to increase with economic development. Importantly, PCOS impacts not only women's health but also has a generalized effect on many aspects of life. At present, the etiology of PCOS is not completely understood, and this condition may represent the consequence of the interaction between genetic and environmental factors [2], including family history, low birth weight, obesity, poor dietary habits, and sedentary lifestyle. According to the American Society for Reproductive Medicine (ASRM) 2018 Guidelines, the first-line treatment of PCOS is lifestyle adjustment, including diet control and exercise, with the weight control being especially important for PCOS patients [3]. Recently, dietary interventions have been reported to ameliorate clinical symptoms of PCOS, including menstrual disorders, and abnormal hormonal indicators and ovulation [4, 5]. Therefore, modification of diet appears as a critical therapeutic modality capable of improving the clinical symptoms of PCOS.

A low-carbohydrate diet (LCD) refers to a dietary structure that helps to manage or prevent disease by limiting the consumption of carbohydrates and correspondingly increasing the intake of proteins and/or lipids [6]. Low-carbohydrate diet has been demonstrated to effectively decrease body weight and facilitate the treatment of infertility in obese PCOS patients [7, 8]. However, the evidence of the actual effect of LCD on the improvement of clinical symptoms of PCOS is lacking. Therefore, the goal of the current study was a systematic review of investigations addressing the impact of the LCD intervention on phenotypic changes in PCOS patients.

## 2. Methods

### 2.1. Search Strategy and Selection of Studies

The search for relevant publications included the Cochrane Library, PubMed, Embase, Cumulative Index of Nursing and Allied Health Literature (CINAHL), China Biomedical Abstracts Database (SinoMed), China Academic Journals Network Publishing Library (CNKI), Wanfang Database, and unpublished grey literature. The search covered the time from the publication of the oldest articles in the respective library to December 2018. The search was performed according to the PICO format [6] and used the following components (see Table 1): participants (P), intervention (I), control (C), outcome (O), and study design (S). The MeSH terms such as “polycystic ovary syndrome” and “low carbohydrate diet” were employed. The search algorithm was constantly adjusted and improved by trial and error approach, taking into account the retrieval requirements specific for each database. The final search strategy included English terms “PCOS/polycystic ovary syndrome” and “Carbohydrate-Restricted/low carbohydrate/low-CHO” and Chinese terms equivalent to “polycystic ovary syndrome/polycystic/PCOS” and “low sugar/low carbon.” The lists of references included in all retrieved articles were reviewed to identify additional potential publications that were not identified by the electronic searches.

### 2.2. Inclusion and Exclusion Criteria

The inclusion criteria were as follows: (1) the study represented original research; (2) the study was designed as a randomized control trial (RCTs); (3) the full text of the publication could be obtained; (4) the target population were women with PCOS; (5) PCOS diagnosis was based on the 2003 Rotterdam criteria; (6) the intervention group was on a low-carbohydrate diet in which carbohydrates accounted for less than 45% of the three major nutrients, and the control group was on a regular diet (carbohydrates accounted for approximately 45%); and (7) availability of all raw data obtained in the trials for the primary and secondary indicators utilized in the current meta-analysis.

Exclusion criteria were as follows: (1) combination of the LCD with other drugs, such as metformin; (2) data duplicated in conference papers and journal articles or in Chinese and English literature. The higher-quality source was selected in these instances; (3) in case of the came content being published in two articles, only one was selected; (4) the publication was an abstract, and a full-text version was not available after contacting the author; (5) the patients were clomiphene citrate-resistant, or infertility was due to causes other than PCOS; and (6) the study was a review.

### 2.3. Data Extraction

Two of the authors (XZ and YZ) conducted the literature search. The differences encountered were resolved by consensus or discussion with the corresponding authors (YG and ZL). The extracted data comprised the name of the first author, year and country of publication, diagnostic criteria, population, sample size, age, study design, duration of the study, intake ratio of the three major nutrients, type of intervention, and endocrine and metabolic indicators, such as body mass index (BMI), HOMA-IR, TC, LDL-C, HDL-C, testosterone (T), sex hormone-binding globulin (SHBG), follicle-stimulating hormone (FSH), and luteinizing hormone (LH).

### 2.4. Quality Assessment

The Cochrane Collaboration Risk of Bias tool was used to assess the risk of bias of the included RCTs. The assessment was based on the information related to the following domains: random sequence generation, allocation concealment, blinding of participants and outcome assessment, incomplete outcome data, selective outcome reporting, and other bias. For each study, the risk of bias was assessed as low, unclear, and high. Any disagreements during the selection process were addressed by discussion until a consensus was reached.

### 2.5. Data Analysis and Synthesis

The data presented in the selected papers were analyzed using the Review Manager (Version 5.3). Weighted mean difference (WMD) or standardized mean difference (SMD), 95% confidence interval (CI), and odds ratio (OR) or relative risk (RR) were used to define the magnitude and statistical significance of the effect. Before the meta-analysis was performed, the heterogeneity between the results of each included study was tested by chi-square test. In the absence of statistical heterogeneity (*P* > 0.1, *I*^2^ < 50%), the fixed effect model was used for analysis, while in its presence (*P* < 0.1, *I*^2^ > 50%), the random effects model was applied.

## 3. Results

### 3.1. Selection of Studies

**Figure 1:**
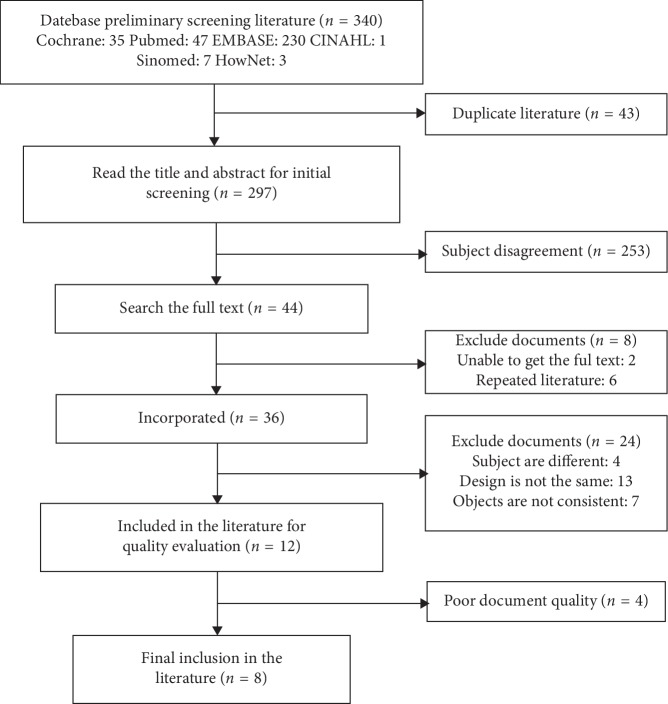
Flowchart of literature search, review process, and selection of studies.

**Figure 2:**
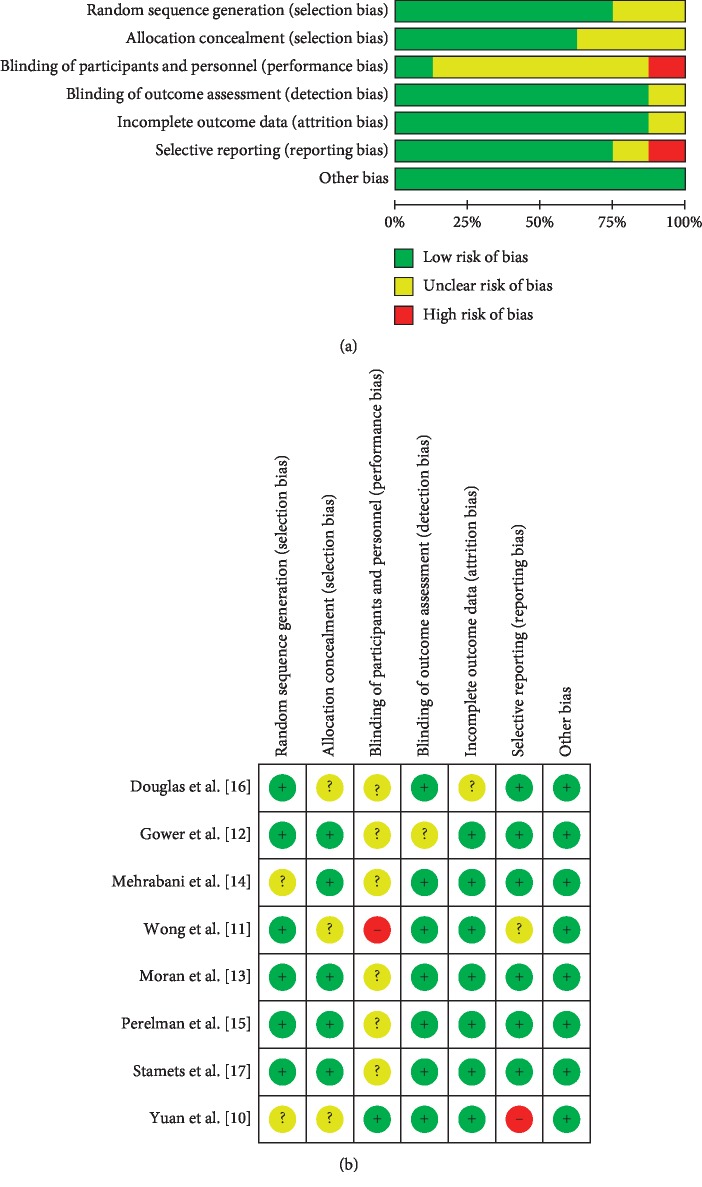
Graphical representation of the risk of bias in the selected studies.

A total of 340 publications were identified in the searched databases (PubMed: 47; Cochrane: 35; Embase: 230; CINAHL:1; Sinomed:7; and HowNet:3), among which 43 were duplicated articles. After browsing the titles and abstracts, 253 were removed because of the subject disagreement. The remaining 44 studies were examined in detail to assess their eligibility. Among these 44 studies, two were excluded because they were unable to get the full text from the rest studies, six were excluded because they were duplicated publications, four were excluded because the subjects were using medications besides the diet, seven were excluded because the measured outcomes did not match our selection, and thirteen were excluded because they were not designed as RCTs. As a result, eight articles [7–14] which met the inclusion criteria were qualified for the meta-analysis (Figure 1 and Table 2). The risk of bias present in the selected 8 studies is illustrated in Figure 2.

### 3.2. Publication Bias

Funnel chart and Egger's regression tests indicated no significant publication bias for meta-analyses assessing the effect of low-carbohydrate diet on PCOS (*t* = 0.02, *P*=0.982).

### 3.3. The Effects of LCD on BMI

**Figure 3:**
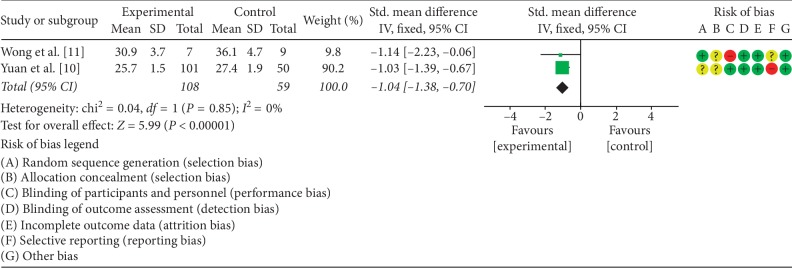
Meta-analysis of the effect of LCD on BMI in PCOS patients.

Two studies [10, 11] analyzed the effect of LCD on the BMI of PCOS patients. The experimental and control groups included a total of 167 patients. There was no statistical heterogeneity between the two studies (*P*=0.85, *I*^2^ = 0%); therefore, the fixed effect model was used to combine the data. Meta-analysis demonstrated that the difference in the BMI between the two groups was statistically significant (SMD = −1.04, 95% CI (−1.38, −0.70), *P* < 0.00001). A summary of the performed meta-analysis is shown in Figure 3.

### 3.4. The Effects of LCD on HOMA-IR

**Figure 4:**
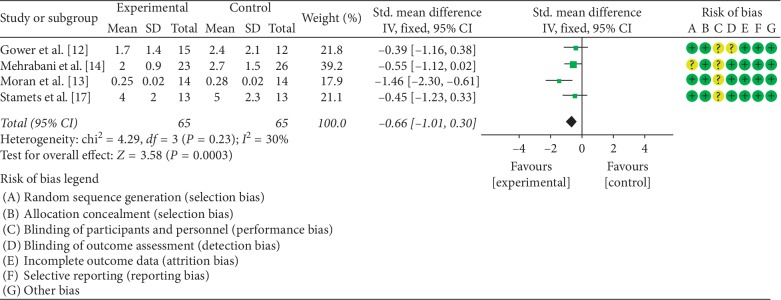
Meta-analysis of the effect of LCD on HOMA-IR in PCOS patients.

The impact of LCD on HOMA-IR was examined in four studies [12–14, 17]. A total of 130 of PCOS patients participated in these investigations. There was no statistical heterogeneity among the four studies (*P*=0.23, *I*^2^ = 30%); therefore, the fixed effect model was used to combine the data. Meta-analysis indicated a statistically significant difference between patients on LCD and control diet (SMD = −0.66, 95% CI (−1.01, −0.30), *P*=0.0003). A summary of the performed meta-analysis is shown in Figure 4.

### 3.5. The Effects of LCD on Endocrine Hormones

#### 3.5.1. The Effects of LCD on FSH

**Figure 5:**
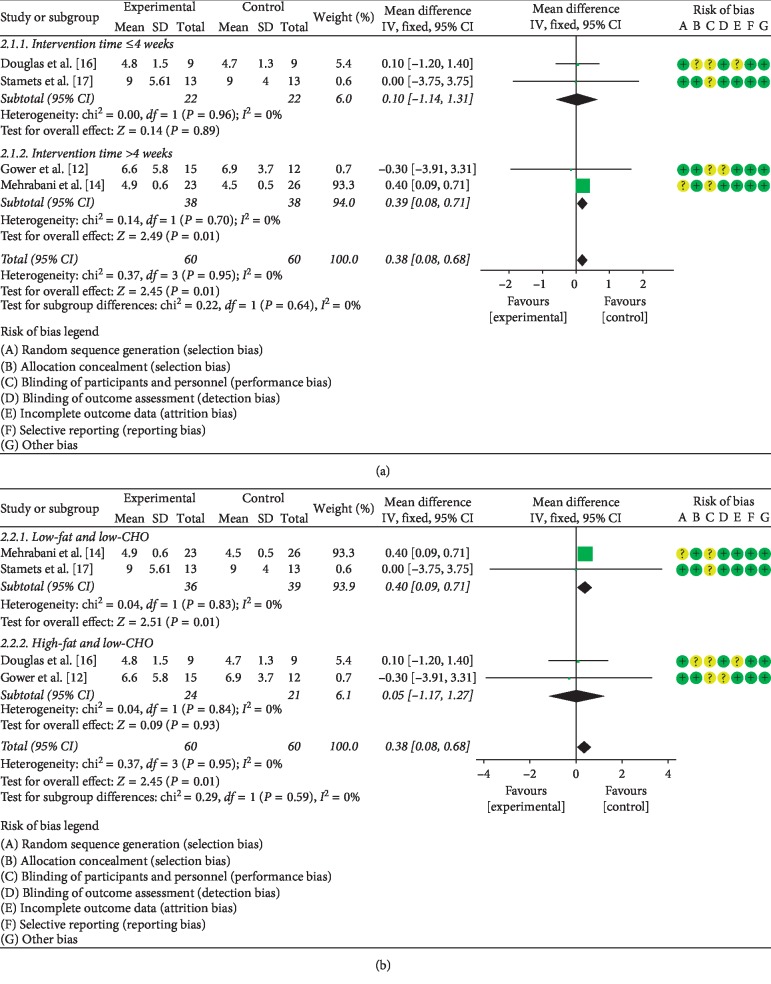
Meta-analysis of the effect of (a) LCD duration on FSH levels in PCOS patients and (b) LCD type on FSH levels in PCOS patients.

Four studies addressing the effect of the LCD on FSH [12, 14, 16, 17] were identified. A total of 120 PCOS patients were enrolled in this research. Since no statistical heterogeneity was detected among the four investigations (*P*=0.95, *I*^2^ = 0%), the fixed effect model was used to pool the data. Meta-analysis indicated a statistically significant difference between patients on LCD (regardless of the diet type and duration) and control diet (MD = 0.38, 95% CI (0.08, 0.68), *P* < 0.05). In addition, given that the type and duration of LCD may influence hormone levels, a subgroup analysis was performed. The stratified analysis documented that LCD intervention longer than 4 weeks had increased the level of FSH more than intervention lasting 4 weeks or less (MD = 0.39, 95% CI (0.08, 0.71), *P* < 0.05) (Figure 5(a)). Moreover, the low-fat/low-CHO LCD (fat <35%, CHO <45%) had significantly higher effect on FSH levels than the high-fat/low-CHO LCD (fat >35%, CHO <45%) (MD = 0.40, 95% CI (0.09, 0.71), *P* < 0.05) (Figure 5(b)).

#### 3.5.2. The Effects of LCD on LH

**Figure 6:**
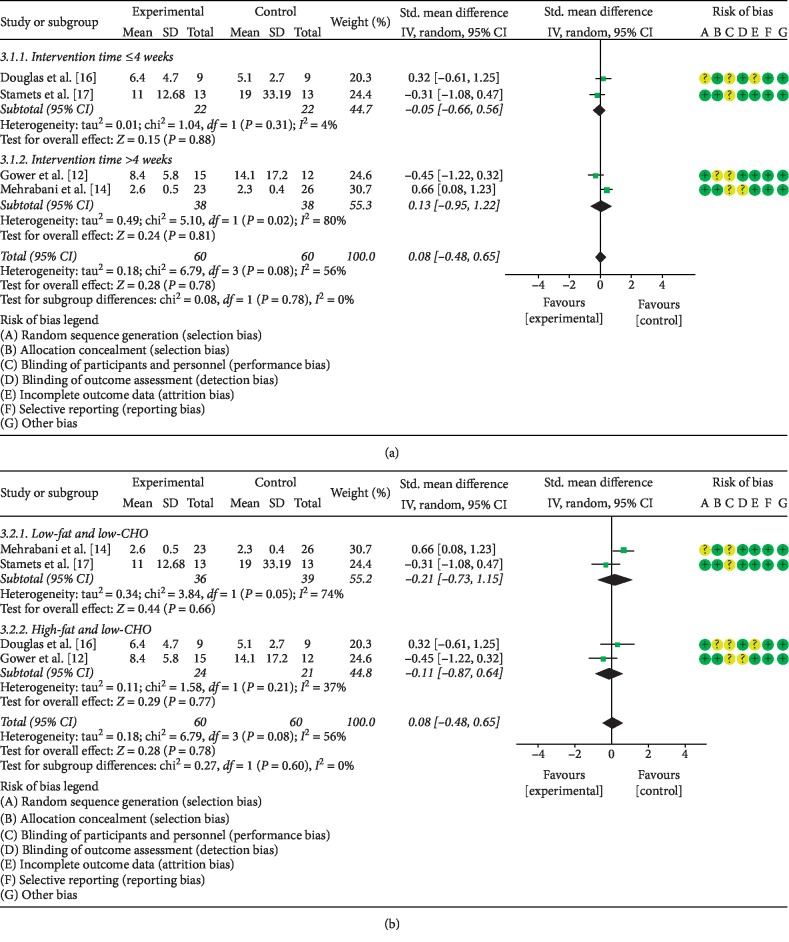
Meta-analysis of the effect of (a) LCD duration on LH levels in PCOS patients and (b) LCD type on LH levels in PCOS patients.

The same four studies addressed the effect of LCD on LH levels in PCOS patients. Given the statistical heterogeneity among the studies (*P*=0.08, *I*^2^ = 56%), the random effects model was utilized to combine the data. Meta-analysis showed that the difference between the PCOS patients subjected to LCD and control PCOS patients was not statistically significant (SMD = 0.08, 95% CI (−0.48, 0.65), *P* > 0.05). In stratified analyses, the effect of LCD intervention longer than 4 weeks on LH levels did not differ significantly from that of shorter LCD interventions (SMD = 0.13, 95% CI (−0.95, 1.22), *P* > 0.05) (Figure 6(a)). Moreover, the effects of low-fat/low CHO LCD was not statistically different from that of high-fat/low CHO diet (MD = 0.21, 95% CI (−0.73, 1.15), *P* > 0.05) (Figure 6(b)).

#### 3.5.3. The Effects of LCD on SHBG

**Figure 7:**
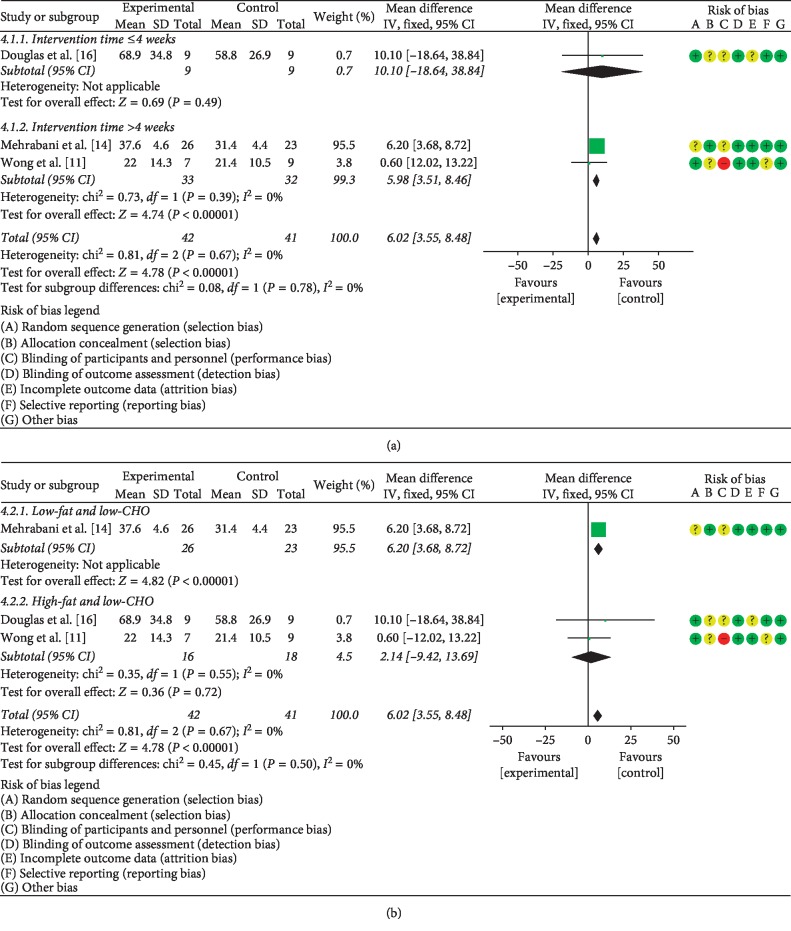
Meta-analysis of the effect of (a) LCD duration on SHBG levels in PCOS patients and (b) LCD type on SHBG in PCOS patients.

Three studies [11, 14, 16] which together included a total of 83 PCOS patients compared the effect of LCD on the level of SHBG. Statistical heterogeneity was detected among these investigations (*P*=0.67, *I*^2^ = 0%), necessitating the use of the fixed effects model to pool the data. Meta-analysis showed a significant difference between PCOS patients treated with LCD intervention and control PCOS patients (MD = 6.02, 95% CI (3.55, 8.48), *P* < 0.05). In stratified analysis, LCD intervention longer than 4 weeks had a higher effect on SHBG levels than intervention lasting 4 weeks or less (MD = 5.98, 95% CI (3.51, 8.46), *P* < 0.05) (Figure 7(a)). Additionally, the low-fat/low-CHO LCD had more pronounced on SHBG levels than the high-fat/low-CHO LCD (MD = 6.20, 95% CI (3.68, 8.72), *P* < 0.05) (Figure 7(b)).

#### 3.5.4. The Effects of LCD on Testosterone

**Figure 8:**
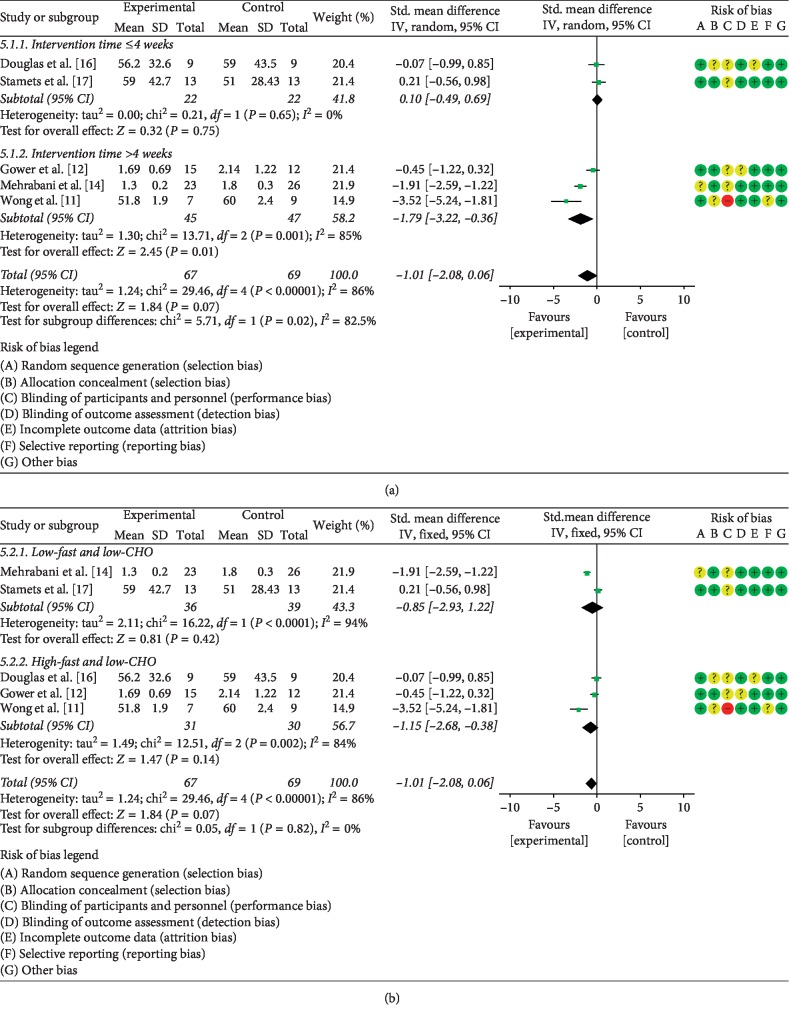
Meta-analysis of the effect of (a) LCD duration on *T* levels in PCOS patients and (b) LCD type on *T* levels in PCOS patients.

The effect of the LCD on testosterone was reported in five publications [11, 12, 14, 16, 17]. These studies involved a total of 136 PCOS patients. Since statistical heterogeneity was present among the studies (*P* < 0.05, *I*^2^ = 86%), the random effects model was employed to combine the data. This meta-analysis did not detect significant difference between the LCD and control groups (SMD = −1.01, 95% CI (−2.08, 0.06), *P* > 0.05). In stratified analysis, LCD intervention longer than 4 weeks had a higher impact on *T* levels than intervention lasting 4 or less weeks (SMD = −1.79, 95% CI (−3.22, −0.36), *P* < 0.05) (Figure 8(a)). Stratified analysis also indicated that the effect of low-fat/low-CHO LCD on *T* levels did not differ significantly from that of the high-fat/low-CHO LCD (SMD = −0.85, 95% CI (−2.93, 1.22), *P* > 0.05) (Figure 8(b)).

#### 3.5.5. The Effects of LCD on Blood Lipids

**Figure 9:**
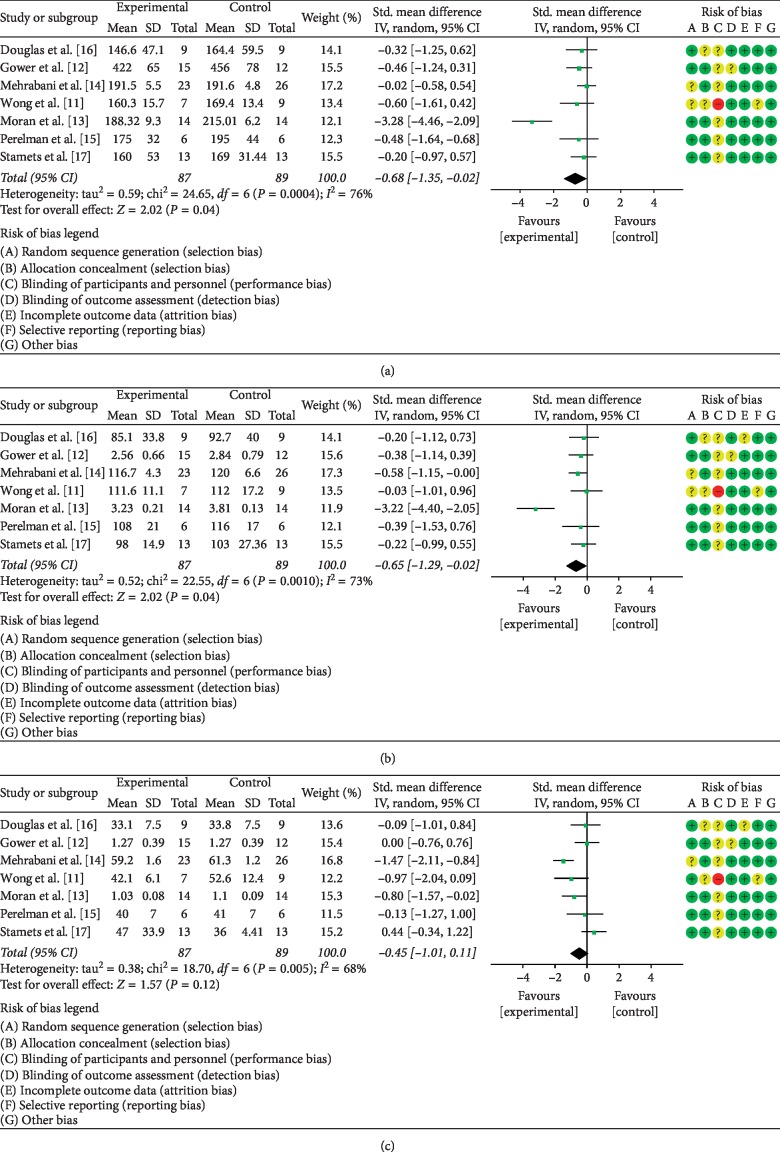
Meta-analysis of the effect of LCD on blood lipids in PCOS patients. (a), TC; (b), LDL-C; (c), HDL-C.

The impact of LCD on blood TC levels in PCOS patients was analyzed in seven studies [11–17] which together enrolled 176 patients. Statistical heterogeneity was present among the investigations (*P*=0.0004, *I*^2^ = 76%); therefore, the data were pooled using the random effects model. As illustrated in Figure 9(a), meta-analysis demonstrated a statistically significant difference between patients treated by LCD and the control group (SMD = −0.68, 95% CI (−1.35, −0.02), *P* < 0.05).

The same seven publications reported the data on the effect of LCD on LDL-c [11–17]. Given the statistical heterogeneity among these studies (*P*=0.0008, *I*^2^ = 74%), the random effects model was used to combine the data. Meta-analysis demonstrated that the difference between the experimental and control groups was statistically significant. (SMD = −0.66, 95% CI (−1.30, −0.02), *P* < 0.05). This analysis is illustrated in Figure 9(b).

Finally, the seven studies [11–17] determined the impact of LCD on the concentration of circulating HDL-C in the same cohort of 176 subjects. The random effects model was used to pool the data since statistical heterogeneity was present among the studies (*P*=0.005, *I*^2^ = 68%). By meta-analysis, the difference between the LCD-treated patients and the control group was not statistically significant (SMD = −0.45, 95% CI (−1.01, −0.11), *P* > 0.05). For details of meta-analysis, see Figure 9(c).

## 4. Discussion

PCOS is a common endocrine disorder in women. It is characterized by abnormal insulin metabolism and high levels of androgen [14, 16]. The cause of PCOS remains to be identified, and effective treatment is not available. The therapy of PCOS is mostly symptomatic and requires long-term management. According to the American Society for Reproductive Medicine (ASRM) 2018 Guidelines, the first-line treatment of PCOS is lifestyle adjustment, including diet control and increased amount of exercise [3]. LCD refers to a dietary structure that manages the symptoms or prevents disease by reducing or limiting the intake of carbohydrates to less than 45% of the total daily calorie intake [18]. LCD may effectively control body weight in overweight or obese people, lower insulin levels, and improve insulin resistance and other endocrine deficiencies [18–21]. Currently, inconsistent data regarding the influence of LCD are present in the published literature, necessitating further studies and analyses of the impact of this nutritional modification on PCOS patients. The present investigation reviewed relevant studies in order to conclude whether LCDs can improve the clinical symptoms associated with PCOS better than traditional or high-carbohydrate diets.

The meta-analysis performed here indicates that LCD can significantly reduce BMI and serum levels of TC and LDL-C in PCOS patients. Moreover, stratified analyses documented that LCD intervention, in particular, the low-fat/low-CHO (less than 35% of fat and less than 45% CHO) and the long-term (more than 4 weeks) LCD can significantly increase the levels of FSH and SHBG, and decrease the level of testosterone in PCOS patients. Therefore, LCD, especially the low-fat/low-CHO LCD and long-term LCD, appears to be efficacious as an adjuvant treatment for PCOS-related manifestations.

The available evidence supports the notion that LCD can effectively control body weight in overweight and obese people, lower insulin levels, and improve insulin resistance and other endocrine system deficiencies [18–22]. The possible mechanism of these beneficial actions involves an LCD-induced decrease in the levels of circulating insulin and glucose [21]. Studies have shown that changes in the circulating levels of IGF-1, insulin-like growth factor binding protein 1 (IGFBP1), glucose, and insulin are typical results of diet control, and these modifications may play a critical function in regulating aging and metabolic homeostasis [23]. LCD regimens reducing the levels of IGF-1, IGFBP1, glucose, and insulin may have beneficial effects on ovarian function. Given the paramount importance of insulin receptor and compensatory hyperinsulinemia in the induction of androgen excess in PCOS women, LCD may also improve hyperandrogenism-related symptoms [24]. In addition, weight loss is associated with a decrease in adipose tissue and thus may negatively modulate the conversion of androgens in estrone. By this mechanism, LCD may reduce the hypothalamic and hypophyseal dysregulation, which underlies the subfertility in PCOS women.

The performed meta-analysis demonstrated that LCD significantly improves insulin resistance. A growing amount of evidence points to insulin resistance and secondary hyperinsulinemia as critical factors in the development of hyperandrogenism, maintenance of metabolic alterations, and anovulation or irregular menstrual cycles in both obese and lean PCOS patients [23]. Studies have shown that excessive concentrations of insulin can cause hyperandrogenism by promoting ovarian and adrenal glands to secrete androgen, inhibiting SHBG synthesis in the liver. Additionally, hyperinsulinemia can prevent follicular development and ovulation. This effect can be mediated by two distinct mechanisms. The first one involves direct block of immature follicles, the arrest of sinus follicular development, while the second mechanism indirectly causes the ovarian response to endogenous gonadotropin, such as an excess of androgen, resulting in an increase in the ratio of luteinizing hormone (LH) to follicle-stimulating hormone (FSH) [13, 24–29]. Based on these findings, most of current treatment strategies aim at the reduction of insulin resistance in PCOS patients, alleviating compensatory hyperinsulinemia, and improving metabolic and ovulatory dysfunction. Accordingly, recent guidelines for women with PCOS and metabolic abnormalities recommend insulin-sensitizing drugs to improve the response to insulin, with the goal of improving fertility.

This meta-analysis showed that long-term and low-fat/low-CHO LCD could restore, at least in part, insulin sensitivity in PCOS patients, counteracting glucose metabolism impairment, gonadotropin imbalance, and ovarian dysfunction. One of the potential mechanisms by which LCD improves these dysfunctions involves the regulation of inositol metabolism. Inositol is one of the nine stereoisomers of cyclohexanol and belongs to the vitamin B family. The most common of cyclohexanol isomeres are myoinositol (MI) and D-chiro-inositol (DCI). The vast majority of intracellular pools of MI are converted into DCI by an NAD/NADH-dependent isomerase, which is inhibited in PCOS due to insulin dysfunction. The imbalance between MI and DCI plays an important role in IR, most likely due to the impaired conversion of MI into DCI. Therefore, the dysregulation of inositol metabolism may lead to decreased insulin sensitivity, hyperinsulinemia, inhibition of the maturation of follicles, and the development of PCOS [30]. LCD may increase insulin sensitivity in PCOS patients by restoring the balance of inositol, thereby improving insulin resistance, decreasing the level of androgen, and restoring the regularity of the menstrual cycle and quality of oocytes in patients with PCOS.

According to the PCOS Guidelines issued by the American Reproductive Medicine Association (ASRM) in 2018, the implementation of a healthy lifestyle is the first-line management method for all PCOS patients. Modification of the diet, adequate exercise, and effective weight loss, all play an important role in PCOS treatment [3]. However, for women with a definitive diagnosis of PCOS and associated reproductive system symptoms, endocrine disorders, or high risk of developing PCOS, the use of medications is necessary, in addition to the healthy lifestyle, to alleviate the clinical symptoms. Combined oral contraceptive (COC), a first-line pharmaceutical treatment for PCOS, can effectively reduce androgen levels and restore normal menstrual cycle. Additionally, it is also conceivable to use metformin and/or inositol to increase insulin sensitivity, and to induce ovulation with letrozole or a selective estrogen receptor modulator (SERM) [3]. Possibly, laparoscopic ovarian diathermy (LOD) or low dose FSH stimulation could be introduced in the case of SERM and/or letrozole resistance [31]. However, the abovementioned treatments promoting ovulation may lead to an increased risk of ovarian hyperstimulation syndrome (OHSS) in PCOS patients, in particular, those with high levels of anti-Müllerian hormone (AMH) [23, 30, 32]. It has been demonstrated that PCOS patients with high AMH levels are not only insensitive to the therapeutic effect of ovulation induction but also have a greater risk of OHSS [31–34].

Therefore, based on the existing PCOS treatment strategies and the conclusions of the present meta-analysis, we suggest that the most important therapy for PCOS patients, especially those with high AMH levels, is long-term and low-fat/low-CHO LCD intervention and the medical treatment utilizing mostly metformin and/or inositol to increase insulin sensitivity. The use of medications inducing ovulation and their dosage for PCOS patients with high AMH level requires further clinical research before evidence-based decisions can be made. In vitro fertilization may be considered in case of combined infertility or resistance to other treatments.

The limitations of this meta-analysis include the relatively small number of relevant studies retrieved and potential differences in the quality of the included publications. Additionally, some of the studies indicated increased exercise activity during the LCD treatment, which might, to some extent, increase the degree of bias. Thus, although LCD may be effective in improving selected clinical symptoms in PCOS patients, because of the small number of high-quality RCTs performed worldwide, definitive conclusions cannot be reached yet. Furthermore, rigorously designed and high-quality, large-scale research on the impact of LCD on PCOS symptoms is necessary to provide solid and reliable evidence to be used in clinical practice.

## 5. Conclusion

Dietary habits are the major cause of the metabolic syndrome, and modification of the diet represents the easiest to achieve preventive measure for this condition. LCD, especially long-term and low-fat/low CHO can effectively reduce body weight and improve the manifestations of the metabolic syndrome such as high blood sugar, insulin resistance, and abnormal lipid metabolism. The meta-analysis presented here demonstrates that LCD treatment in women with PCOS has significantly improved BMI, lipid levels (TC, and LDL-C), HOMA-IR, T, FSH, and SHBG.

The relationship between carbohydrate diet and metabolic syndrome is extremely complex. At present, there is no consensus regarding a unified theory, and further research is warranted. However, since proper control of carbohydrate intake in daily diet has defined effects on the prevention and treatment of metabolic syndrome, dietary interventions represent an important approach to improve the clinical symptoms of PCOS patients.

## Figures and Tables

**Table 1:** Inclusion criteria.

P (participants)	Women with a diagnosis of polycystic ovary syndrome

I (intervention)	The intervention group was on a low-carbohydrate diet (carbohydrate accounted for less than 45% of the three major nutrients)

C (comparisons)	The control group was on a regular diet (carbohydrates accounted for more than 45%)

O (outcomes)	The change in body mass index (BMI), homeostatic model assessment of insulin resistance (HOMA-IR), total cholesterol (TC), low-density lipoprotein cholesterol (LDL-C), high-density lipoprotein cholesterol (HDL-C), follicle-stimulating hormone (FSH), luteotropic hormone (LH), total testosterone (T), and sex hormone-binding globulin (SHBG)

S (study type)	Randomized controlled trials (RCTs) assessing the effects of low-carbohydrate diet on PCOS

**Table 2:** Characteristics of the included studies.

Study	Country	Sample size		Diet composition (protein : fat : CHO) (%)		Relevant outcomes	Mean age (years)		Mean BMI (baseline)		Duration
		Trial group	Control group	Trial group	Control group		Trial group	Control group	Trial group	Control group	
Yuan et al. [10]	China	101	50	40–45 : 30–35 : 20–25	25–30 : 30 : 45	BMI	28.6 ± 1.7	28.5 ± 9.5	27.7 ± 1.7	27.6 ± 1.8	8 weeks
Mehrabani et al. [14]	Australia	23	26	30 : 30 : 40	15 : 30 : 55	T, FSH, LH, SHBG, HOMA-IR, TC, HDL-C, LDL-C	30.5 ± 6.4	28.5 ± 5.2	31.9 ± 4.0	31.1 ± 4.6	12 weeks
Gower et al. [12]	USA	15	12	19 : 40 : 41	18 : 27 : 55	T, FSH, LH, HOMA-IR, TC, HDL-C, LDL-C	31.2 ± 5.8		31.8 ± 5.7		8 weeks
Moran et al. [13]	USA	14	14	30 : 30 : 40	15 : 30 : 55	HOMA-IR, TC, HDL-C, LDL-C	32 ± 1.2	33 ± 1.2	37.9 ± 1.6	37.7 ± 1.9	16 weeks
Wong et al. [11]	USA	7	9	20 : 35 : 45	20 : 25 : 55	BMI, T, SHBG, TC, HDL-C, LDL-C	15.4 ± 1.3	16.3 ± 2.2	36.2 ± 5.3	33.9 ± 4.7	24 weeks
Perelman et al. [15]	USA	6	6	15 : 45 : 40	15 : 25 : 60	TC, HDL-C, LDL-C	30 ± 7		39 ± 7		3 weeks
Douglas et al. [16]	USA	9	9	15 : 45 : 43	16 : 31–33 : 55–56	T, FSH, LH, SHBG, TC, HDL-C, LDL-C	33 ± 6		30.0 ± 3.7		1 weeks
Stamets et al. [17]	USA	13	13	30 : 30 : 40	15 : 30 : 55	T, FSH, LH, HOMA-IR, TC, HDL-C, LDL-C	29 ± 4	26 ± 4	38 ± 4	37 ± 5	4 weeks
